# Chemotherapeutic control of Gram-positive infection in white sea bream (*Diplodus sargus*, Linnaeus 1758) broodstock

**DOI:** 10.14202/vetworld.2019.316-324

**Published:** 2019-02-23

**Authors:** Nadia G. M. Ali, Ibrahim M. Aboyadak, Heba S. El-Sayed

**Affiliations:** 1 Fish Disease Laboratory, National Institute of Oceanography and Fisheries, Egypt; 2 Fish Reproduction Laboratory (Marine Hatchery), National Institute of Oceanography and Fisheries, Egypt

**Keywords:** *Bacillus cereus*, histopathology, high-performance liquid chromatography, oxytetracycline, sensitivity, *Staphylococcus epidermidis*

## Abstract

**Aim:**

This study aimed to identify the pathogenic bacteria responsible for the septicemic disease affecting white sea bream brooders and determining the sensitivity of the recovered isolates to different antibiotics followed by estimation of long-acting oxytetracycline (OTC) efficacy in controlling this disease, and finally, determining the proper dose regimen.

**Materials and Methods:**

Biolog microbial identification system was used for determination of the pathogens which are responsible for this disease. Agar disk diffusion test and minimum inhibitory concentration (MIC) were used to determine the antibiotic susceptibility of recovered isolates. Oxytetracycline (OTC) was used at a dose level of 100 mg/kg body weight for the treatment of diseased fish, and the OTC concentration in the serum samples was determined by high-performance liquid chromatography.

**Results:**

Fifteen *Staphylococcus epidermidis* and 11 *Bacillus cereus* isolates were recovered from the lesion of muscle, tail, eye, and heart blood. *S. epidermidis* isolates were sensitive to OTC, ciprofloxacin, enrofloxacin, spiramycin, erythromycin (E), and florfenicol. *B. cereus* isolates were sensitive to all mentioned antibiotics except E. Based on the MIC test, all *B. cereus* isolates were sensitive to OTC with MIC ranging between <0.125 and 4 µg/ml and 11 *S. epidermidis* isolates were sensitive with MIC ranging between <0.125 and 8 µg/ml, while four isolates were resistant. Different degrees of degenerative changes were present in the hepatopancreas, posterior kidney, eye, and skin tissues of diseased fish.

**Conclusion:**

Single intraperitoneal injection of long-acting OTC at a dose of 100 mg/kg body weight was effective in termination of *S. epidermidis* and *B. cereus* infection in white sea bream (*D. sargus*) broodstock.

## Introduction

Aquaculture is one of the fastest growing animal production sectors worldwide, where farmed finfish production rate has increased from 27.6 to 52.3 million (nearly duplicated) over the past decade from 2005 to 2015 [1]. Aquaculture is considered as an imperative choice to provide cheap animal protein for the growing world population. The global fishery production for human consumption reached 76 million tons in 2015. The aquaculture production is expected to exceed the fishery production by 1.36% fold at 2025 [2].

Expansion in marine aquaculture is an urgent priority for the Egyptian government, so it launched the largest maricultural farm in the Middle East at Berket Ghalioun. It targeted to culture about 5460 hectares with different marine fish species, and so there is a need for diversification and introduction of new species. White sea bream (*Diplodus sargus*) is considered a good candidate for aquaculture as it has a high commercial value and good acceptability by consumers [3]. It is also an omnivorous fish and, therefore, needs fewer nutritional requirements in comparison to other Sparidae [4].

Bacterial diseases are the most dominant pathogens that are responsible for severe diseases and outbreaks in marine-cultured fish [5,6]. Most of the bacterial disease etiological agents are considered a part of the normal flora that is present in water, stress condition as bad water quality, inadequate diet, overcrowding, and frequent handling during artificial spawning are considered to be predisposing factors of lowering fish immunity and subsequently initiating disease conditions [7,8]. *Streptococcus* spp., *Lactococcus garvieae, Staphylococcus epidermidis*, and *Micrococcus luteus* are among the most common Gram-positive bacterial infection of cultured marine fish [9,10], while vibriosis is the most Gram-negative bacterial infection [11-13].

Route of drug administration is a limiting factor that plays a considerable role in the success or failure of a treatment process, as it greatly affects the drug pharmacokinetic parameters including absorption, distribution, biotransformation, and excretion [14]. Intraperitoneal and intravenous drug administration provides not only the maximum bioavailability but also allows the drug to reach the site of infection very rapidly achieving high serum level [15], as well. Drug formulation has a vital role in the dosage regimen (frequency of application), i.e., long-acting preparations provide sustainable drug release in treated animal serum, which allow long-lasting effect that may extend for several days [16].

Broodstocks are extremely valuable and expensive fish. It requires a long time and effort for preparation; loss of any brooder fish is equal to the loss of thousands of produced fry each season, and so control of broodstock diseases is considered a priority for sustainable aquaculture.

This study aimed to identify the pathogenic bacteria responsible for the septicemic disease affecting white sea bream brooders and determining the sensitivity of the recovered isolates to different antibiotics followed by estimation of long-acting oxytetracycline (OTC) efficacy in controlling this disease, and finally, determining the proper dose regimen.

## Materials and Methods

### Ethical approval

Fish used in the current research were handled, transported, examined, and treated following the guidelines of the National Advisory Committee for Laboratory Animal Research (NACLAR) [17] and CCAC [18] regarding the care and use of fish in research, teaching, and testing which were approved by the National Institute of Oceanography and Fisheries (NIOF) Ethical Committee, Egypt.

### Study location

The present work was conducted in the marine hatchery, NIOF, Alexandria, located at longitude 31°12’44.8” N and latitude 29°53’05.1”E.

### Fish specimens

Sixty white sea bream (*D. sargus*, Linnaeus 1758) broodstocks were examined through clinical examination. Of them, 23 were dead and moribund fish were used for postmortem, bacteriological, and histopathological examination. 20 clinically diseased fish were used in the treatment trial, and 10 healthy fish were used for the determination of antibiotic concentration in their serum. Fish ranged between 210 and 587 g in body weight and 16-28 cm in total length.

### Antemortem and postmortem inspection

Antemortem and postmortem examinations were done as described by Stoskopf [19] for the detection of any changes in fish behavior or appearance also for the determination of any gross abnormalities in internal organs.

### Isolation of bacterial etiological agent

It was performed as described by Aboyadak *et al*. [20], in which five samples were taken from each fish (tail lesions, muscle lesions, eye lesions if present, hepatopancreas, and heart blood). Each sample was individually inoculated to Tryptic Soy Broth for 12 h, then streaked on tryptic soy agar (Oxoid^®^), and incubated at 33°C for 18-24 h.

### Identification of the isolated strains using biolog system

Few colonies from each recovered isolate were smeared on a glass slide and stained with Gram stain following the procedures mentioned by Black and Black [21].

Biolog microbial identification system (Biolog Inc., Hayward, CA, USA) is an automated, accurate, and rapid identification method that is based on carbon source utilization assay as each microorganism has a phenotypic fingerprint. GEN III MicroPlate has 12 columns each of eight rows: The first nine columns are for 71 carbon source utilization assays and the rest 3 columns for 23 chemical sensitivity assays. The utilization of carbon sources and resistance to the inhibitory chemicals were determined through tetrazolium redox dye.

A surface area of about 3 mm in diameter from the pure isolate that was grown on Tryptic Soy Agar was picked up using a cotton-tipped inoculator’s swab. The swap was transferred and suspended in a special clean tube containing inoculating fluid. The turbidity was measured and adjusted to 95% using turbidimeter. After that, the bacterial cell suspension was poured into Biolog GEN III Microplates using the multichannel pipette. The inoculated microplate was covered with its lid and placed for incubation, and the result of the monitoring was logged in the OmniLog system.

### Histopathological examination

The histopathological examination was carried out according to Suvarna *et al*. [22]. Tissue specimens from the hepatopancreas, posterior kidney, skin, and eye were fixed in 10% buffered formalin, dehydrated in ascending grade ethyl alcohol and cleared in xylene, sectioned to 4-µm thickness, mounted over a glass slide, and then stained with hematoxylin and eosin (H and E). Stained tissue sections were examined and photographed using Optika Microscope with a digital camera (Optika, Italy).

### Antibiotic susceptibility test

Agar disk diffusion test was done to determine the sensitivity of the recovered bacterial isolates to OTC (OTC 30 µg), ciprofloxacin (CIP 5 µg), enrofloxacin (ENR 5 µg), florfenicol (F 10 µg), spiramycin (SP 100 µg), and erythromycin (E 15 µg) according to CLSI [23].

### Determination of the minimum inhibitory concentration (MIC) of OTC

It was determined for all recovered isolates using broth macrodilution test according to Hack *et al*. [24].

### Preparation of OTC standard solution

About 10.66 µl was pipetted from Alamycin 300^®^ and made up to 1000 µl with sterile distilled water to achieve a final concentration of 3.2 mg/1000 µl, and then, a double-fold serial dilution was done for 10 consecutive dilutions.

### Procedures

The overnight growth of each bacterial isolate on tryptic soy broth was diluted and adjusted to 0.5 McFarland standard. After that, 0.5 ml was added to 99.5 ml sterile Mueller Hinton Broth, then 4.9 ml was added to each of 11 sterile screw-caped, numbered test tubes followed by addition of 100 µl from previously prepared OTC standard solution to the corresponding test tube to achieve a final concentration of 64, 32, 16, 8, 4, 2, 1, 0.5, 0.25, and 0.125 µg/ml, while the last tube was left without antibiotic as control. The prepared test tubes were incubated at 33°C for 24 h. MIC was determined as the lowest concentration at no visible bacterial growth.

### Treatment experiment

#### Drug

Alamycin 300^®^ (Norbrook Co., United Kingdom) long-acting solution contains OTC dihydrate 30 mg/ml.

#### Fish grouping

Twenty clinically diseased *D. sargus* were randomly divided into two equal groups, each containing 10 fish; Group 1 was treated with Alamycin 300 at a dose of 100 mg/kg body weight by intraperitoneal injection, and Group 2 remains as a control. Each group was kept in 2000-L fiberglass tank supplied with marine water 35‰ with continuous aeration. The temperature was thermostatically controlled at 25°C and fish were observed for 14 days from the first dose.

#### Determination of OTC in fish serum by high-performance liquid chromatography (HPLC)

Ten healthy *D. sargus* were used in this experiment; each fish was inoculated with Alamycin 300^®^ at a dose of 100 mg/kg body weight. Fish were kept in a fiberglass tank at which water temperature was thermostatically adjusted at 25°C, and then 500 µl blood was collected through the caudal vessels from each fish at 24, 48, 72, 96, 120, 144, and 168-h post-injection. The serum was separated by centrifuge at 5000 rpm and then was stored at −23°C. OTC concentration was determined in serum samples according to the method described by Lei *et al*. [25] using Nexera X2 HPLC system, Shimadzu, Japan, and C18 reverse-phase column, Zorbax SB-C18 5.0 µm, 4.6 mm×250 mm, Agilent, USA. The mobile phase consisted of 0.01 mol/l oxalic acid, methanol, and acetonitrile with a volume ratio of 83:7:10, respectively. OTC level was detected at 355-nm wavelength.

## Results

### Antemortem and postmortem inspection

**Figure-1:**
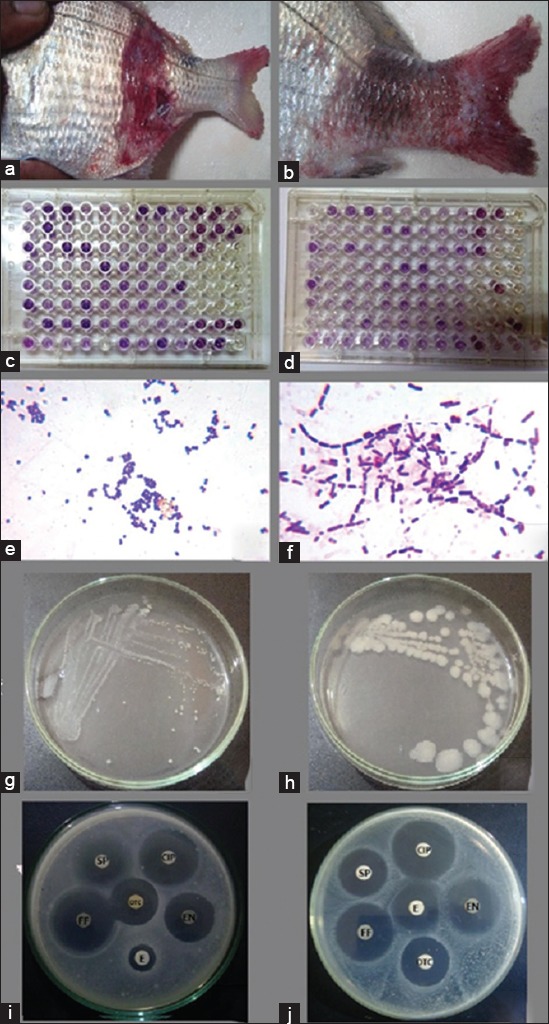
(a and b) Naturally infected white sea bream (*Diplodus sargus*) showing scale desquamation, skin ulceration even complete loss of skin, and appearance of musculature with congestion and hemorrhage in caudal peduncle together with tail erosion and hemorrhages. (c and d) Biolog GEN III microplate showing the biochemical profile of *Staphylococcus epidermidis* (c) and *Bacillus cereus* (d). (e) Gram-stained *S. epidermidis* appeared as Gram-positive cocci, 0.65-0.91 µm in diameter, present as single, pairs, and clusters. (f) Gram-stained *B. cereus* appeared as Gram-positive spore-forming long bacilli, 4.97-7.48 µm in length and 1.58-1.64 in width, arranged in short or long chains. (g) *S. epidermidis* colonies on Tryptic Soy Agar appeared as white pinpoint colonies about 0.2-1 mm in diameter.)h) *B. cereus* colonies on Tryptic Soy Agar appeared as large white granular colonies with irregular perimeters about 1.5-5 mm in diameter. (i) Antibiogram indicated the sensitivity of *B. cereus* to oxytetracycline (OTC), ciprofloxacin (CIP), enrofloxacin (ENR), florfenicol (F), and spiramycin (SP), while it resists erythromycin (E). (j) Antibiogram indicated the sensitivity of *S. epidermidis* to OTC, CIP, ENR, F, SP, and E.

The inspections show hemorrhagic skin ulcers of different degrees (starting with scale desquamation, hyperemia, followed by deep hemorrhagic skin ulcers reaching the deep musculature), unilateral exophthalmia, fin erosions, and hemorrhagic tail with severe erosions (Figure-1a and b). Congested hepatopancreas and kidney were the most prominent PM lesion. The recorded mortality was estimated by 38.33% (23 of 60 fish).

### Results of bacterial isolation and identification

Twenty-six Gram-positive isolates were recovered from nine moribund fish; 15 isolates were cocci, and 11 were bacilli (Table-1).

**Table-1:** Number of *Staphylococcus epidermidis* and *Bacillus cereus* isolates from each organ of diseased white sea bream (*Diplodus sargus*) broodstock.

Organ of isolation	Number of *Staphylococcus epidermidis* isolates	Number of *Bacillus cereus* isolates
Muscle	5	5
Eye	1	1
Heart	4	0
Tail	5	5
Total	15	11

Biolog microbial identification system illustrates the presence of 15 *S. epidermidis* (Figure-1c) and 11 *Bacillus cereus* isolates (Figure-1d).

#### Culture characters of the recovered bacteria

*S. epidermidis* appeared as white pinpoint colonies about 0.2-1 mm in diameter (Figure-1g), while *B. cereus* appeared as large white granular colonies with irregular perimeters about 1.5-5 mm in diameter (Figure-1h).

#### Morphological characteristics of the recovered bacteria

*S. epidermidis* appeared as Gram-positive cocci ranged between 0.65 and 0.91 µm in diameter, and it was present as single, pairs, and clusters (Figure-1e), while *B. cereus* appeared as Gram-positive rods present in short or long chains, and each bacillus was 4.97-7.48 µm in length and 1.58-1.64 in width (Figure-1f).

*S. epidermidis* was isolated from the heart, tail, muscle, and eye, while *B. cereus* isolate was isolated from the tail, muscle, and eye as shown in Table-1.

### Results of the histopathological examination

**Figure-2:**
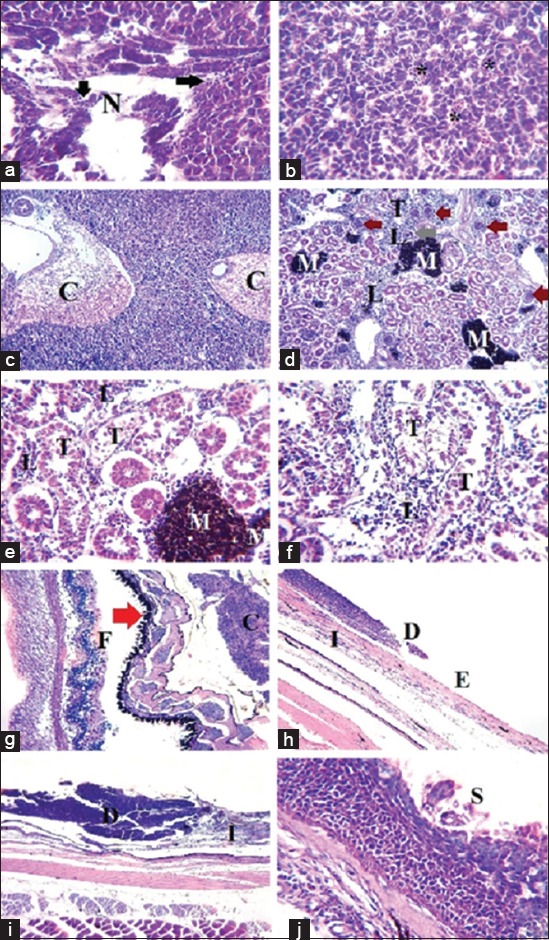
Histopathological lesions induced by *Staphylococcus epidermidis* and *Bacillus cereus* infection in affected white sea bream (*Diplodus sargus*). (a-c) Hepatopancreas showing loss of normal tissue architecture, presence of necrotic areas (N) with mononuclear cell infiltration (arrow), congested hepatic sinusoids (*) and congested distended blood vessels (C), hematoxylin and eosin (*H and E*), X=400 in (a and b) and 100 in (c). (d-f) Posterior kidney showing glomerular hypertrophy with narrow Bowman’s space (brown arrow), degenerated shrinkage glomerular taught (gray arrow), detached tubular epithelium (T), interstitial mononuclear cell infiltration (L), melanomacrophage centers activation (M), *H and E*, X=100 in (d) and 100 in (e and f). (g) Eye with marked separation between retina layers, especially, pigment epithelium layer (red arrow) which also is corrugated and the photoreceptor layer (F), (C) is the choroid body, *H and E*, X=100. (h-j) Skin showing destruction of epidermis even complete loss (D) with exposure of dermis (E) and presence of leukocytic infiltration (I), in less affected cases loss of the superficial layer of the stratified squamous epithelium (S), *H and E*, X=100 in (h and i) and 400 in (j).

The histopathological study of naturally infected *D. sargus* tissues revealed the presence of different pathological alterations (Figure-2). Hepatopancreas tissue lost their normal architecture with the presence of inflammatory reaction manifested in mononuclear cell infiltration, necrotic areas were also present, hepatic tissue congestion expressed as congested hepatic sinusoids, and distended blood vessels engorged with erythrocytes (Figure-2a-c). Posterior kidney tissue was severely affected as glomerular hypertrophy with narrowed Bowman’s space was present. Some degenerated shrinkage glomerular tuft was also observed. Furthermore, renal tubules were involved in this degenerative changes in which detachment of tubular epithelium was observed. Inflammation of renal tissue was presented as interstitial mononuclear cell infiltration and melanomacrophage center activation (Figure-2d-f). Exophthalmic tissue examination indicated marked separation between retina layers, especially pigment epithelium and the photoreceptor layers, as these tissues appeared corrugated (Figure-2g). Ulcerated skin lesions show destruction of epidermis that was completely lost with exposure of dermis and presence of leukocytic infiltration, but, in less affected cases, the superficial layer of the stratified squamous epithelium was eroded (Figure-2h-j).

### Antibiotic sensitivity test results

*S. epidermidis* was sensitive to OTC, CIP, ENR, F, SP, and E (Figure-1j), while *B. cereus* was sensitive to all mentioned antibiotics except E (Figure-1i) as presented in Table-2.

**Table-2:** Antibiotics susceptibility test results for *Staphylococcus epidermidis* and *Bacillus cereus.*

Antibiotic	*Staphylococcus epidermidis*		*Bacillus cereus*	
	
	Inhibition zone mm	Interpretation	Inhibition zone mm	Interpretation
OTC 30 μg	22	Susceptible	21	Susceptible
CIP 5 μg	27	Susceptible	26	Susceptible
ENR 5 μg	24	Susceptible	22	Susceptible
F 10 μg	24	Susceptible	27	Susceptible
SP 100 μg	21	Susceptible	17	Susceptible
E 15 μg	19	Susceptible	11	Resistant

OTC=Oxytetracycline, CIP=Ciprofloxacin, ENR=Enrofloxacin, F=Florfenicol, SP=Spiramycin, E=Erythromycin

### The MIC of OTC

Twenty out of 26 isolates resembling 9 *S. epidermidis* and all the 11 *B. cereus* were highly susceptible to OTC with MIC ranged between <0.12 and 4 µg/ml, while two *S. epidermidis* isolates were intermediately susceptible with MIC equal to 8 µg/ml, but four isolates were resistant with MIC equal to 16-32 µg/ml (Table-3).

**Table-3:** MIC of oxytetracycline.

Bacterial strain	Isolate Number	MIC of oxytetracycline μg/ml	Interpretation
*Staphylococcus epidermidis*	1	<0.125	Susceptible
	2	16	Resistant
	3	32	Resistant
	4	8	Intermediate
	5	32	Resistant
	6	0.5	Susceptible
	7	32	Resistant
	8	8	Intermediate
	9	4	Susceptible
	10	<0.125	Susceptible
	11	4	Susceptible
	12	0.125	Susceptible
	13	0.5	Susceptible
	14	0.125	Susceptible
	15	4	Susceptible
*Bacillus cereus*	1	<0.125	Susceptible
	2	<0.125	Susceptible
	3	2	Susceptible
	4	<0.125	Susceptible
	5	1	Susceptible
	6	1	Susceptible
	7	1	Susceptible
	8	1	Susceptible
	9	4	Susceptible
	10	0.25	Susceptible
	11	0.25	Susceptible

Interpretation: Susceptible ≤4, Intermediate=8, Resistant ≥16 μg/ml. MIC=Minimum inhibitory concentration

### Results of treatment trial

Treated fish showed gradual improvement starting with disappearance of hyperemia and hemorrhage from tail and skin ulcers at the 2^nd^-day post-treatment followed by partial healing of ulcers throughout the 1^st^ week. The cumulative mortality during 2 weeks after treatment was 40% in infected non-treated group but was only 10% in the treated group as one severely ulcerated fish died at the 2^nd^ day post-treatment.

### OTC level in fish serum following intraperitoneal inoculation

OTC level in *D. sargus* serum reached 34.57±1.09, 25.98±0.96, 20.39±1.2, 13.10±0.32, 10.04±0.26, 7.68±0.42, and 5.14±0.23 (µg/ml) at 24, 48, 72, 96, 120, 144, and 168 h, respectively, following the intraperitoneal injection as presented in Table-4.

**Table-4:** Oxytetracycline levels in the serum of white sea bream (*Diplodus sargus*) broodstock at different time points post-treatment.

Sampling time (h postinjection)	Oxytetracycline level (μg/ml)
24	34.57±1.09
48	25.98±0.96
72	20.39±1.2
96	13.10±0.32
120	10.04±0.26
144	7.68±0.42
168	5.14±0.23

n=6, values are mean±standard error

## Discussion

Introduction of a new species is an essential element for expansion in marine culture; White Sea bream (*D. sargus*) is an excellent candidate for this purpose as it has good acceptability for consumers; and increased fry (seeds) production is an essential element for sustainable aquaculture.

Streptococci considered a potential cause of diseases in mariculture [26]. *S. epidermidis* is considered a potential fish pathogen as it can lead to localized, as well as systemic, infection in cultured fresh and marine fish species [27-29]. Infections with *B. cereus* were also recorded by Baya *et al*. [30] and Chandra *et al*. [31] in striped bass and stinging catfish, respectively.

In the current study, *D. sargus* broodstock suffered from increased mortality that was estimated by 38.33%. Diseased fish showed deep skin ulcers that reached the musculature, hemorrhages, fin and tail erosions, and exophthalmia with congested parenchymatic organs (hepatopancreas and posterior kidney), and similarly, anti- and post-mortem lesions were recorded by Golomazou *et al*. [3] in diseased caged white sea bream. Kubilay and Uluköy [32] isolated *S. epidermidis* from diseased gilthead sea bream (*Sparus aurata*) with hemorrhages on gills, skin, and fins, and Kusuda and Sugiyama [33] isolated *S. epidermidis* from diseased yellowtail *(Seriola quinqueradiat*a) and red sea bream *(Chrysophrys majo*r) that showed exophthalmia, swelling, and congestion of caudal peduncle, these observations were identical to the present work findings. Chandra *et al*. [31] reported nearly similar signs in stinging catfish. *Heteropneustes fossilis* infected with *B. cereus*. Parallel to the current findings Austin [34] cited the presence of generalized necrotizing dermatitis in *B. cereus-*infected fish. Aboyadak *et al*. [7] recorded similar clinical signs and postmortem lesions in sea bass infected with *B. cereus* and *S. epidermidis*.

The histopathological examination indicated the presence of some pathological lesions involving hepatopancreas as inflammation, congestion, and necrosis. Posterior kidney showed degenerated glomeruli and renal tubules with inflammatory cell aggregation. These findings were supported by Austin [34], who reported the presence of focal necrosis and petechial hemorrhages in the liver and kidney of *B. cereus*-infected fish. Marked separation between retina layers especially pigment epithelium and the photoreceptor layer of affected eye which could have resulted from the increased intraocular pressure.

The ante- and post-mortem findings together with the histopathological lesions indicated the severity of the current infection affecting *D. sargus* broodstock and it is mainly induced by different virulence factors produced by the isolated causative agents, especially in stressed broodstock that suffered from frequent handling and hormonal treatment (injection) which is accompanied with scales detachment and skin abrasions that act as a porter of entry for pathogenic and opportunistic bacteria.

*S. epidermidis* is not considered a part of normal fish flora as it has many virulence factors responsible for its pathogenicity. They are mainly extracellular products as proved by Huang *et al*. [27], who found that inoculation of tilapia with filter-sterilized culture broth of *S. epidermidis* causes similar mortality as bacterial suspension contains 1.34×10^9^ CFU/ml. Namvar *et al*. [35] mentioned some virulence factors of *S. epidermidis* as biofilm formation, polysaccharide intercellular adhesion, surface adhesion protein, poly-γ-glutamic acid (responsible for phagocytosis inhibition), toxins as staphylococcal enterotoxin-like toxin, and delta toxin. Pinheiro *et al*. [36] recorded that *S. epidermidis* exhibits high toxigenic potential as they identified eight enterotoxin genes from 85 clinical isolates.

*B. cereus* has many virulence factors, including hemolysin, which have a dermonecrotic and vascular permeability activity [37]. Prasad [38] isolated enterotoxigenic *B. cereus* from 10 marine fish guts. *B. cereus* is known to produce substances responsible for virulence including hemolysins (I, II, and III), enterotoxins, cytotoxin K, and phospholipase which in combination induce the disease condition [39]. *B. cereus* is famous as foodborne poisoning microorganism, but it is also considered the causative agent of many other serious diseases such as endocarditis, ocular infection, myonecrosis and cutaneous infections, nosocomial infections, meningitis, and urinary tract infection [40-44].

*S. epidermidis* grown as white pinpoint colonies (0.2-1 mm in diameter), morphologically present as Gram-positive cocci 0.65-0.91 µm in diameter which was identical to that described by Huang *et al*. [27] and Kusuda and Sugiyama [33] for *S. epidermidis* isolated from clinically diseased fish.

*B. cereus* colonies were large white granular with irregular perimeters, about 1.5-5 mm in diameter, *B. cereus* appeared as Gram-positive spore-forming long bacilli, 4.97-7.48 µm in length and 1.58-1.64 in width, arranged in short or long chains that are similar to the description reported by Bottone [45].

The recovered *S. epidermidis* isolates were sensitive to CIP, ENR, F, SP, and E, but only four isolates resisted OTC. These results are in complete harmony with Kusuda and Sugiyama [33] as they reported its sensitivity to OTC, chloramphenicol, and E, but it partially agrees with that of Kubilay and Uluköy [32] as they recorded *S. epidermidis* sensitivity to CIP, ENR, and chloramphenicol, while it was resistant to OTC and E. Sergelidis *et al*. [46] recorded the sensitivity of *S. epidermidis* isolated from fish to quinolones and its resistance to E.

*B. cereus* isolates were sensitive to OTC, CIP, ENR, F, and SP, but they resisted E. This result was compatible with that done by Chandra *et al*. [31] except for OTC. Weber *et al*. [47] also reported the sensitivity of *B. cereus* to chloramphenicol, CIP, and tetracycline. The mentioned difference in antibiotic susceptibility profile of *S. epidermidis* and *B. cereus* isolates could be attributed to the presence of certain antibiotic resistance genes.

OTC is currently available and approved by the U.S. Food and Drug Administration for use as a chemotherapeutic agent in food fish [48]. Intraperitoneal administration of long-acting OTC at a dose of 100 mg/kg body weight was very effective in termination of the present infection as the mortality rate decreases from 40% in infected non-treated group to 10% in infected treated group, together with disappearance of hemorrhage and congestion followed by regenerative changes as ulcer healing, regrowth of eroded fins, and tail. Only one fish died in the treated group, which may have resulted from the presence of large ulcers on its body before treatment. It was severely affected by bacterial invasion and toxins or due to osmoregulation failure.

The successfulness of the treatment is not only reflecting the proper selection of antibiotic, but also the drug formulation has a role as long-acting formula administrated once (each fish handled once to decrease the stress and ulceration during manipulation), as well. Parenteral administration has many advantages over oral drug administration: Diseased fish became off-food, on the other hand, some interfering substances which may present in water as ions can inactivate, chelate, or decrease the concentration of the orally administrated drug.

Sensitivity test indicated the high susceptibility of *S. epidermidis* and *B. cereus* isolates to OTC, also, the low MIC accelerates the response to the treatment, although there were four *S. epidermidis* isolates having MIC of 16 and 32 µg/ml. That means these isolates resist OTC, but the treatment was successful, and this can be explained by increasing the serum concentration over 32 µg/ml, and at this point, OTC became effective.

Serum OTC level reaches 34.57±1.09 µg/ml at 24 h post-drug administration, and then, it remains over 5 µg/ml on the 7^th^ day, which indicates the efficacy of single intraperitoneal inoculation in protection and treatment against susceptible pathogens for at least 7 consecutive days. Nearly similar result was recorded by Bowden [49], who found that OTC remains over 4 µg/ml after 168-h post-intramuscular or intraperitoneal administration in yellow perch (*Perca flavescens*), while Rigos *et al*. [16] determined the 24-h OTC plasma concentration as 18 µg/ml after intramuscular administration of 50 mg/kg in healthy grouper (*Epinephelus marginatus*) which differs from our findings, due to the difference in the used dose.

## Conclusion

Single intraperitoneal injection of long-acting OTC at a dose of 100 mg/kg body weight was effective in termination of *S. epidermidis* and *B. cereus* infection in white sea bream (*D. sargus*) broodstock.

## Authors’ Contributions

NGMA: designed the study, performed clinical and postmortem examination and bacterial isolation and identification, also performed the histopathological examination and wrote the manuscript. IMA: designed and conducted the treatment trial, sensitivity test, and HPLC analysis and helped in manuscript writing and preparations. HSE: fish sampling, rearing, and observation. All authors read and approved the final manuscript.
